# Supplementary material for: Dig out, Dig in! Plant-based diet at the Late Bronze Age copper production site of Prigglitz-Gasteil (Lower Austria) and the relevance of processed foodstuffs for the supply of Alpine Bronze Age miners
Source: PLoS One. 2021 Mar 24;16(3):e0248287. doi: 10.1371/journal.pone.0248287 (PMC7990284; doi:10.1371/journal.pone.0248287)
Supplement: Attachment — Submitted filename: Response to Reviewers.docx [file pone.0248287.s002.docx]

PONE-D-20-28652
Dig Out, Dig In! The plant-based diet of Late Bronze Age miners at the copper production site of Prigglitz-Gasteil (Lower Austria), and a few general thoughts on archaeological remains of processed food
PLOS ONE

Dear Dr. Heiss,

Thank you for submitting your manuscript to PLOS ONE. After careful consideration, we feel that it has merit but does not fully meet PLOS ONE’s publication criteria as it currently stands. Therefore, we invite you to submit a revised version of the manuscript that addresses the points raised during the review process.

All comments must be fully addressed before re-submission.

Please submit your revised manuscript by Dec 19 2020 11:59PM. If you will need more time than this to complete your revisions, please reply to this message or contact the journal office at [plosone@plos.org](mailto:plosone@plos.org). When you're ready to submit your revision, log on to <https://www.editorialmanager.com/pone/> and select the 'Submissions Needing Revision' folder to locate your manuscript file.

Please include the following items when submitting your revised manuscript:

- A rebuttal letter that responds to each point raised by the academic editor and reviewer(s). You should upload this letter as a separate file labeled 'Response to Reviewers'.
- A marked-up copy of your manuscript that highlights changes made to the original version. You should upload this as a separate file labeled 'Revised Manuscript with Track Changes'.
- An unmarked version of your revised paper without tracked changes. You should upload this as a separate file labeled 'Manuscript'.

If you would like to make changes to your financial disclosure, please include your updated statement in your cover letter. Guidelines for resubmitting your figure files are available below the reviewer comments at the end of this letter.

If applicable, we recommend that you deposit your laboratory protocols in protocols.io to enhance the reproducibility of your results. Protocols.io assigns your protocol its own identifier (DOI) so that it can be cited independently in the future. For instructions see: <http://journals.plos.org/plosone/s/submission-guidelines#loc-laboratory-protocols>

We look forward to receiving your revised manuscript.

Kind regards,

Peter F. Biehl, PhD
Academic Editor
PLOS ONE
Additional Editor Comments:

Your manuscript has now been seen by a referee, whose comments are appended below. You will see from these comments that while the referees find your work of potential interest, it has raised substantial concerns that must be addressed. In light of these comments, we cannot accept the manuscript for publication, but would be interested in considering a revised version that addresses these serious concerns.

Journal Requirements:

When submitting your revision, we need you to address these additional requirements.

1. Please ensure that your manuscript meets PLOS ONE's style requirements, including those for file naming. The PLOS ONE style templates can be found at

<https://journals.plos.org/plosone/s/file?id=wjVg/PLOSOne_formatting_sample_main_body.pdf> and

<https://journals.plos.org/plosone/s/file?id=ba62/PLOSOne_formatting_sample_title_authors_affiliations.pdf>

We have carefully re-checked the requirements and hope that our manuscript meets PLOS One’s style requirements.

2. We note that Figures 2 and 3 in your submission contain map/satellite images which may be copyrighted. All PLOS content is published under the Creative Commons Attribution License (CC BY 4.0), which means that the manuscript, images, and Supporting Information files will be freely available online, and any third party is permitted to access, download, copy, distribute, and use these materials in any way, even commercially, with proper attribution. For these reasons, we cannot publish previously copyrighted maps or satellite images created using proprietary data, such as Google software (Google Maps, Street View, and Earth). For more information, see our copyright guidelines: <http://journals.plos.org/plosone/s/licenses-and-copyright>.

We require you to either (1) present written permission from the copyright holder to publish these figures specifically under the CC BY 4.0 license, or (2) remove the figures from your submission:

2.1.    You may seek permission from the original copyright holder of Figures 2 and 3 to publish the content specifically under the CC BY 4.0 license.

We recommend that you contact the original copyright holder with the Content Permission Form (<http://journals.plos.org/plosone/s/file?id=7c09/content-permission-form.pdf>) and the following text:

“I request permission for the open-access journal PLOS ONE to publish XXX under the Creative Commons Attribution License (CCAL) CC BY 4.0 (<http://creativecommons.org/licenses/by/4.0/>). Please be aware that this license allows unrestricted use and distribution, even commercially, by third parties. Please reply and provide explicit written permission to publish XXX under a CC BY license and complete the attached form.”

Please upload the completed Content Permission Form or other proof of granted permissions as an "Other" file with your submission.

In the figure caption of the copyrighted figure, please include the following text: “Reprinted from [ref] under a CC BY license, with permission from [name of publisher], original copyright [original copyright year].”

2.2.    If you are unable to obtain permission from the original copyright holder to publish these figures under the CC BY 4.0 license or if the copyright holder’s requirements are incompatible with the CC BY 4.0 license, please either i) remove the figure or ii) supply a replacement figure that complies with the CC BY 4.0 license. Please check copyright information on all replacement figures and update the figure caption with source information. If applicable, please specify in the figure caption text when a figure is similar but not identical to the original image and is therefore for illustrative purposes only.

The following resources for replacing copyrighted map figures may be helpful:

USGS National Map Viewer (public domain): <http://viewer.nationalmap.gov/viewer/>

The Gateway to Astronaut Photography of Earth (public domain): <http://eol.jsc.nasa.gov/sseop/clickmap/>

Maps at the CIA (public domain): <https://www.cia.gov/library/publications/the-world-factbook/index.html> and <https://www.cia.gov/library/publications/cia-maps-publications/index.html>

NASA Earth Observatory (public domain): <http://earthobservatory.nasa.gov/>

Landsat: <http://landsat.visibleearth.nasa.gov/>

USGS EROS (Earth Resources Observatory and Science (EROS) Center) (public domain): [http://eros.usgs.gov/#](http://eros.usgs.gov/)

Natural Earth (public domain): <http://www.naturalearthdata.com/>

The map in Figure 2 was made by the first author using ArcGIS Pro. This fact, alongside with the underlying data, is now explicitly stated in the Methods section.

The aerial photograph in Figure 3 is freely publishable under a CC-BY license. The content permission is now added to the submission.

3. We note that Figure 7 includes an image of a participant in the study.

As per the PLOS ONE policy ([http://journals.plos.org/plosone/s/submission-guidelines#loc-human-subjects-research](about:blank#loc-human-subjects-research)) on papers that include identifying, or potentially identifying, information, the individual(s) or parent(s)/guardian(s) must be informed of the terms of the PLOS open-access (CC-BY) license and provide specific permission for publication of these details under the terms of this license. Please download the [Consent Form for Publication in a PLOS Journal](about:blank) ([http://journals.plos.org/plosone/s/file?id=8ce6/plos-consent-form-english.pdf](about:blank)). The signed consent form should not be submitted with the manuscript, but should be securely filed in the individual's case notes. Please amend the methods section and ethics statement of the manuscript to explicitly state that the patient/participant has provided consent for publication: “The individual in this manuscript has given written informed consent (as outlined in PLOS consent form) to publish these case details”.

If you are unable to obtain consent from the subject of the photograph, you will need to remove the figure and any other textual identifying information or case descriptions for this individual.

We had already obtained informed consent from the person depicted in Figure 7, and the consent form had been properly signed and submitted with the manuscript. As the individual is not a patient but a colleague, there is no such thing as case notes. We are therefore unsure what else to do with the consent form but resubmit it.

We have added the suggested statement to the Methods section in an adapted form.

[Note: HTML markup is below. Please do not edit.]

Reviewers' comments:

Reviewer's Responses to Questions

**Comments to the Author**

1. Is the manuscript technically sound, and do the data support the conclusions?

The manuscript must describe a technically sound piece of scientific research with data that supports the conclusions. Experiments must have been conducted rigorously, with appropriate controls, replication, and sample sizes. The conclusions must be drawn appropriately based on the data presented.

Reviewer #1: Partly

2. Has the statistical analysis been performed appropriately and rigorously?

Reviewer #1: Yes

3. Have the authors made all data underlying the findings in their manuscript fully available?

The [PLOS Data policy](http://www.plosone.org/static/policies.action#sharing) requires authors to make all data underlying the findings described in their manuscript fully available without restriction, with rare exception (please refer to the Data Availability Statement in the manuscript PDF file). The data should be provided as part of the manuscript or its supporting information, or deposited to a public repository. For example, in addition to summary statistics, the data points behind means, medians and variance measures should be available. If there are restrictions on publicly sharing data—e.g. participant privacy or use of data from a third party—those must be specified.

Reviewer #1: Yes

4. Is the manuscript presented in an intelligible fashion and written in standard English?

PLOS ONE does not copyedit accepted manuscripts, so the language in submitted articles must be clear, correct, and unambiguous. Any typographical or grammatical errors should be corrected at revision, so please note any specific errors here.

Reviewer #1: Yes

5. Review Comments to the Author

Please use the space provided to explain your answers to the questions above. You may also include additional comments for the author, including concerns about dual publication, research ethics, or publication ethics. (Please upload your review as an attachment if it exceeds 20,000 characters)

Reviewer #1: It is an interesting article and worth publishing, with valuable new data that can enhance our knowledge and interpretation of the past

Before publishing, I would recommend restructuring the article’s focus from just a report on the findings of a research project, to how the research project findings contribute further to our understanding of Late Bronze Age economy, societies etc. Hence, I suggest the paper is not just about the Prigglitz-Gasteil site, but rather a ‘window’ into the economy and food supply to work forces linked with mining in Late Bronze Age societies of Eastern Alps, Lower Austria, Styria (where it is?- it is a regional name the majority of readers might not be familiar with) and Western Hungary – page 4

Taking these considerations into account, together with comment #5, we have now improved the section “Research goals” to make our intentions more visible, and to present the scope and limitations of the current study. Moreover, the entire manuscript structure has been reorganised. We hope that insufficiently explained thoughts are now more clearly laid out to the reader, and that the overarching questions are now addressed in a better way.

Toponyms have been amended accordingly.

That would alter the text e.g. by deleting or modifying the text on vegetation history of the site or proximity of other Late Bronze Age sites: if such information is not used in the interpretation of the findings, maybe it is redundant? - page 8

We are now referring to local vegetation in the Discussion section, and are generally elaborating on gathered fruit in much greater detail.

The Abstract and Introduction do not reflect the ideas presented in the article, including cuisine, which goes beyond the idea of consumption. Further, the text cannot only focus on the description of findings without linking it into the concepts of cuisine as proposed in the first part of the article.

We have adjusted the manuscript’s title, abstract, and introduction accordingly.

The discussion part has been amended accordingly.

I am not sure how the concept of cuisine is relevant to question posed in this paper, since the question are related to the food provision and consumption.

We think that it makes a difference to include culinary aspects into the *chaîne opératoire* of cereal processing: The information which elements of the various processing stages from harvested sheaves/grain to ready-to-eat dishes allows to gain information on supply chains as it has not been accessible before.

We have now tried to explain this in a better way, and to better highlight cuisine as the “missing link” between crop and consumption.

1) The author needs to make clear that he looks only at the charred material and a small amount of parenchyma. Such remains are very valuable when talking about cuisine if the data is related to other types of plant remains as phytoliths (Saul H, Madella M, Fischer A, Glykou A, Hartz S, et al. (2013) Phytoliths in Pottery Reveal the Use of Spice in European Prehistoric Cuisine. PLoS ONE 8(8): e70583.doi:10.1371journal.pone.0070583) or protein (Hendy, J., Colonese, A.C., Franz, I. et al. Ancient proteins from ceramic vessels at Çatalhöyük West reveal the hidden cuisine of early farmers. Nat Commun 9, 4064 (2018). <https://doi.org/10.1038/s41467-018-06335-6>).

We have looked at the total assemblage of charred plant macroremains and amorphous charred objects (ACO); all ACOs larger than 2 mm were evaluated for any preserved plant tissues, resulting in their classification as cereal products and fruit parenchyma. We hope that now we have been able to explain these issues in a better way.

We are aware of the fact that additional remains will bring additional information, but see that this has not been explained very well. We are aware of the possibilities in proteomics, aDNA, and lipid residual analyses, however, these analyses are within the scope of the follow-up projects. We have now tried to better explain this.

The identified charred silica skeletons of e. g. the millet glumes are indeed phytoliths, although we did not explicitly use this term.

2) It would be useful if the author could acknowledge that he is presenting only part of the picture of what is considered the chain operatoire of a cuisine, since he does not talk about ways of food preparation like baking, cooking, eating raw, or ways of consumption. Fig 22 is not sufficient to answer those questions.

We have now elaborated on the issues the reviewer has raised, and hope that now we explain them sufficiently.

3) There is no discussion on the role of wild plants, e.g. nuts or fruits, in later Bronze Age cuisine.

We have added discussion on these aspects.

4) There is no discussion about the legumes as part of obtaining, preparing and consumption of food stuffs.

We have added discussion on these aspects.

5) Since the focus of the article is related mainly to the charred materia,l I suggest looking at the food as material, as in plant food consumption e.g. Van der Venn, M. 2008. Food as embodied material culture: diversity and change in plant food consumption. Journal of Roman Archaeology 21: 83-109. This would ground the author in the issue of plants as material culture.

We realise that we had taken for granted that plants from archaeological contexts would be considered a part of material culture, and instead immediately focused on less clear issues such as the differentiation between their “ecofacts state” (grains/seeds) and their “artefacts state” (processed foodstuffs, dishes). We have now modified the manuscript accordingly, in order to better explain our intentions. The reference to Marijke van der Veen’s paper has been added.

The part where the data is presented is well structured and written with very good figures illustrating the points made.

In the discussion, I would suggest returning to the idea of cuisine (if it is a concept the author decides to keep), and answer the questions based on the charred and parenchyma data from the Prigglitz-Gasteil site. For example, if and how if so, the diet of the miners was restricted due to the food provisions coming from outside the site? What does the presence of legumes tell us about food production? Were they cultivated in similar a way to cereals or were they grown among cereal crops, and hence appeared in assemblages?

We have now elaborated on all issues raised in the discussion section within the course of the general restructuring of the manuscript.

The wild plant remains are treated in the text as auxiliary data. Their availability during particular seasons, like elder or strawberry, indicate the part of the year work in the mines took place. Moreover, other plants like e.g. sloe, hazel, or wild fruits, indicate other seasons or storage practices as part of the food supply strategy. It is assumed in the text Last but not least: fruits and nuts that wild fruits and nuts were foraged, but by whom, the miners or food suppliers?
Were ‘The most frequent weed taxa belong to members of the goosefoot family (Chenopodiaceae=Amaranthaceae p. p.) and to wild millets (Poaceae-Panicoideae, including e. g. Echinochloa and wild Setaria taxa). These wild millets showed an overall distribution pattern which seemed similar to the one of cultivated millets’ (p22) intentionally gathered? If so what this tell us about food preferences, food supply etc? Further, what does the consumption of cultivated and wild food tells us about Late Bronze Age economy in terms of food production and labour specialisation.

No evidence currently speaks against the miners/metallurgists or other “sur place” craftspeople gathering the wild fruits as well as the antlers in the surrounding woods themselves. We have now added discussion of these considerations to the manuscript.

The overlapping distribution patterns of cultivated and wild millets could possibly indicate their origin from the same fields, and thus a weedy character of the wild millets. The Amaranthaceae p. p. show an inverse pattern (at least when comparing T3 vs. T4). At the current state of analysis, we prefer not to interpret these weedy taxa, as the fine chronology is still missing: Although the overall temporal range of LBA activities is narrow in Prigglitz, the distribution patterns also contain depth in time. A forthcoming publication will deal with the distribution patterns of wild and cultivated species and help clear up these questions.

We have now pointed out these issues and considerations in the manuscript.

6. PLOS authors have the option to publish the peer review history of their article ([what does this mean?](https://journals.plos.org/plosone/s/editorial-and-peer-review-process#loc-peer-review-history)). If published, this will include your full peer review and any attached files.


If you choose “no”, your identity will remain anonymous but your review may still be made public.


**Do you want your identity to be public for this peer review?** For information about this choice, including consent withdrawal, please see our [Privacy Policy](https://www.plos.org/privacy-policy).

Reviewer #1: **Yes:** Liliana Janik

[NOTE: If reviewer comments were submitted as an attachment file, they will be attached to this email and accessible via the submission site. Please log into your account, locate the manuscript record, and check for the action link "View Attachments". If this link does not appear, there are no attachment files.]

While revising your submission, please upload your figure files to the Preflight Analysis and Conversion Engine (PACE) digital diagnostic tool, <https://pacev2.apexcovantage.com/>. PACE helps ensure that figures meet PLOS requirements. To use PACE, you must first register as a user. Registration is free. Then, login and navigate to the UPLOAD tab, where you will find detailed instructions on how to use the tool. If you encounter any issues or have any questions when using PACE, please email PLOS at [figures@plos.org](mailto:figures@plos.org). Please note that Supporting Information files do not need this step.

*In compliance with data protection regulations, you may request that we remove your personal registration details at any time.* [*(Remove my information/details)*](https://www.editorialmanager.com/pone/login.asp?a=r)*. Please contact the publication office if you have any questions.*
